# Acute Infection and Subsequent Subclinical Reactivation of Herpes Simplex Virus 2 after Vaginal Inoculation of Rhesus Macaques

**DOI:** 10.1128/JVI.01574-18

**Published:** 2019-01-04

**Authors:** Ming Lo, Jia Zhu, Scott G. Hansen, Timothy Carroll, Christina Farr Zuend, Laura Nöel-Romas, Zhong-Min Ma, Linda Fritts, Meei-Li Huang, Sijie Sun, Ying Huang, David M. Koelle, Louis J. Picker, Adam Burgener, Lawrence Corey, Christopher J. Miller

**Affiliations:** a Center for Comparative Medicine, University of California Davis, Davis, California, USA; b California National Primate Research Center, University of California Davis, Davis, California, USA; c Department of Laboratory Medicine, University of Washington, Seattle, Washington, USA; d Vaccine and Infectious Diseases Division, Fred Hutchinson Cancer Research Center, Seattle, Washington, USA; e Vaccine and Gene Therapy Institute, Oregon Health & Science University, Beaverton, Oregon, USA; f Oregon National Primate Research Center, Oregon Health & Science University, Beaverton, Oregon, USA; g National HIV and Retrovirology Labs, J.C. Wilt Infectious Disease Research Centre, Public Health Agency of Canada, Winnipeg, Manitoba, Canada; h University of Manitoba, Winnipeg, Manitoba, Canada; i Department of Medicine, University of Washington, Seattle, Washington, USA; j Unit of Infectious Diseases, Department of Medicine Solna, Center for Molecular Medicine, Karolinska Institute, Karolinska University Hospital, Stockholm, Sweden; University of Southern California

**Keywords:** female reproductive tract, T-cell responses, cervix, herpes simplex virus, proteomics, subclinical infection, vaginal swabs

## Abstract

Herpes simplex virus 2 (HSV-2) is a common sexually transmitted infection with a highly variable clinical course. Many infections quickly become subclinical, with episodes of spontaneous virus reactivation. To study host–HSV-2 interactions, an animal model of subclinical HSV-2 infection is needed. In an effort to develop a relevant model, rhesus macaques (RM) were inoculated intravaginally with two or three HSV-2 strains (186, 333, and/or G) at a total dose of 1 × 10^7^ PFU of HSV-2 per animal. Infectious HSV-2 and HSV-2 DNA were consistently shed in vaginal swabs for the first 7 to 14 days after each inoculation. Proteins associated with wound healing, innate immunity, and inflammation were significantly increased in cervical secretions immediately after HSV-2 inoculation. There was histologic evidence of acute herpesvirus pathology, including acantholysis in the squamous epithelium and ballooning degeneration of and intranuclear inclusion bodies in epithelial cells, with HSV antigen in mucosal epithelial cells and keratinocytes. Further, an intense inflammatory infiltrate was found in the cervix and vulva. Evidence of latent infection and reactivation was demonstrated by the detection of spontaneous HSV-2 shedding post-acute inoculation (10^2^ to 10^3^ DNA copies/swab) in 80% of RM. Further, HSV-2 DNA was detected in ganglia in most necropsied animals. HSV-2-specifc T-cell responses were detected in all animals, although antibodies to HSV-2 were detected in only 30% of the animals. Thus, HSV-2 infection of RM recapitulates many of the key features of subclinical HSV-2 infection in women but seems to be more limited, as virus shedding was undetectable more than 40 days after the last virus inoculation.

**IMPORTANCE** Herpes simplex virus 2 (HSV-2) infects nearly 500 million persons globally, with an estimated 21 million incident cases each year, making it one of the most common sexually transmitted infections (STIs). HSV-2 is associated with increased human immunodeficiency virus type 1 (HIV-1) acquisition, and this risk does not decline with the use of antiherpes drugs. As initial acquisition of both HIV and HSV-2 infections is subclinical, study of the initial molecular interactions of the two agents requires an animal model. We found that HSV-2 can infect RM after vaginal inoculation, establish latency in the nervous system, and spontaneously reactivate; these features mimic some of the key features of HSV-2 infection in women. RM may provide an animal model to develop strategies to prevent HSV-2 acquisition and reactivation.

## Abstract (precis)

Herpes simplex virus 2 (HSV-2) infects nearly 500 million persons globally, with an estimated 21 million incident cases each year, making it one of the most common sexually transmitted infections (STIs). HSV-2 is associated with increased human immunodeficiency virus type 1 (HIV-1) acquisition, and this risk does not decline with the use of antiherpes drugs. As initial acquisition of both HIV and HSV-2 infections is subclinical, study of the initial molecular interactions of the two agents requires an animal model. We found that HSV-2 can infect RM after vaginal inoculation, establish latency in the nervous system, and spontaneously reactivate; these features mimic some of the key features of HSV-2 infection in women. RM may provide an animal model to develop strategies to prevent HSV-2 acquisition and reactivation.

## INTRODUCTION

Herpes simplex virus 2 (HSV-2) infection is a common chronic viral sexually transmitted infection (STI) of humans. In 2012, an estimated 417 million people were infected with HSV-2, and nearly 300 million of these were women (1). Further, there were estimated to be 12 million incident cases among women that year (1). HSV-2 was one of the opportunistic infections described in the original report of AIDS (2, 3). In 1988, Stamm et al. reported an increase of human immunodeficiency virus type 1 (HIV-1) infections among men who have sex with men (MSM) with incident and prevalent HSV-2 (4). Extension of these data to female populations throughout the world was noted in the early 1990s, and epidemiologic studies suggest that HSV-2 increases the per-contact rate of HIV-1 acquisition in women 3-fold (5). In regions of high HSV-2 prevalence, it is estimated that 25 to 35% of HIV incidence is directly attributable to HSV-2 infections (5, 6). Subclinical HSV-2 reactivation occurs in over 90% of HSV-2-seropositive persons (7). The immune cell populations that respond to control HSV-2 reactivation in genital tissues express surface receptors for HIV (8), which may explain enhanced HIV acquisition in HSV-2^+^ women.

As both HIV-1 and HSV-2 infections can be acquired subclinically, studies of the molecular interactions of the two agents require an animal model. Mouse models of vaginal HSV-2 infection are available, but unlike human infection, they result in a lethal acute disease (reviewed in reference 9). Moreover, HSV-2 does not spontaneously reactivate in mice. Spontaneous reactivation from latency occurs in dorsal root ganglia of guinea pigs (GP), providing a useful model to study the virology of HSV-2 latency (reviewed in reference 9); however, reagents to study HSV-2 immunopathogenesis in GP are limited.

Simian immunodeficiency virus (SIV) infection in rhesus macaques (RM) is a valuable animal model for HIV pathogenesis studies, but prior attempts to infect RM with HSV have largely failed (10, 11). In fact, RM appear to be less susceptible to HSV infection than some New World primates (10, 11). The relative resistance of RM to HSV-2 infection is likely because TRIM5a expression in RM cells partially blocks HSV-2 replication *in vitro* (12). In 2009, Crostarosa et al. reported that after experimental vaginal HSV-2 inoculation, RM became infected and HSV-2 DNA shedding in genital secretions was subsequently detected (13). Further, enhanced vaginal transmission of simian-human immunodeficiency virus (SHIV) was reported for HSV-2-infected RM without genital lesions (13). This study, while useful conceptually, reported limited data on neuronal latency, the virological characteristics of reactivation, and the immune responses to HSV-2. Thus, the utility of the RM for modeling HSV-2 infection remains unclear (12). The goal of the current study was to characterize HSV-2 infection in RM using the same assays and sampling methods that have been used for humans to provide a much more detailed understanding of HSV-2 infection in this animal model (14–18).

## RESULTS

### Acute HSV-2 infection.

**FIG 1:**
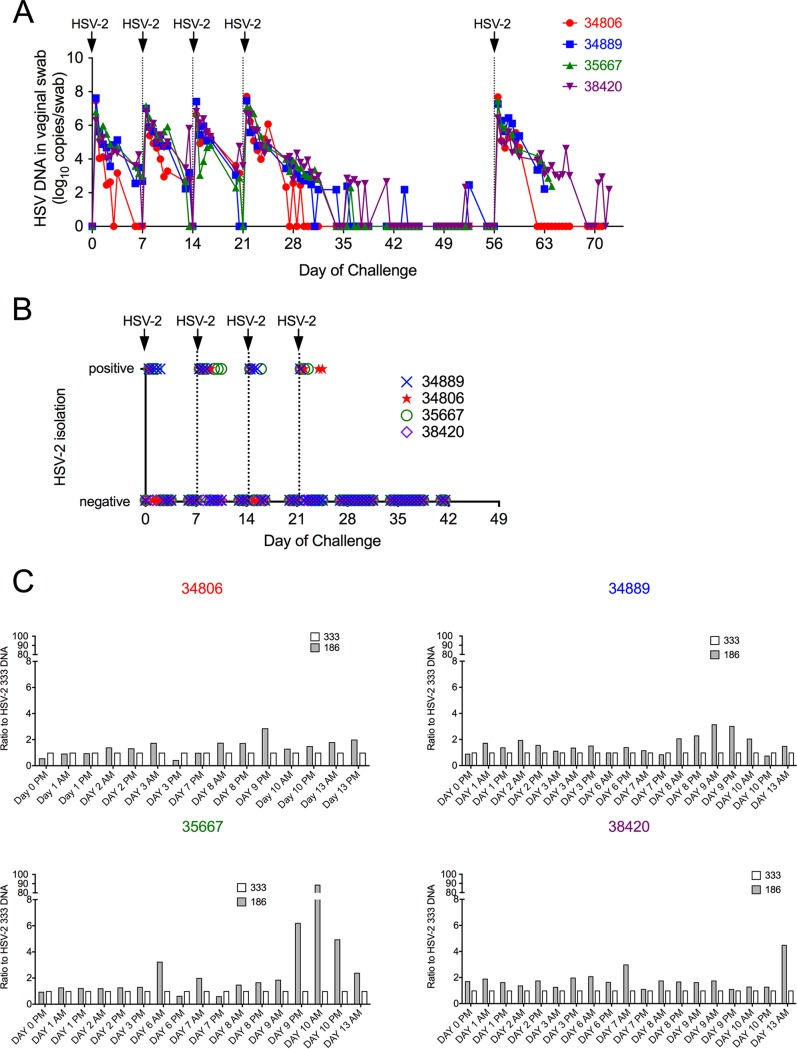
HSV-2 shedding in cervicovaginal secretions of rhesus macaques after HSV-2 inoculation (group 1). Each of the four female rhesus macaques was intravaginally challenged with total 1 × 10^7^ PFU of HSV-2 in 1 ml of inoculum containing a 1:1 mixture of HSV-2 186 and HSV-2 333 once a week for 4 weeks. After a 5-week interval, the animals were reinoculated with the same HSV-2 mixture and necropsied 1 to 2 weeks later. Twice-daily vaginal swabs were collected (except weekends) after the first HSV-2 inoculation. Animal numbers are indicated and color-coded to matched graphed data. (A) Results of PCR to quantitate HSV-2 gB DNA levels in vaginal swabs. (B) Results of attempt to isolate infectious HSV-2 from vaginal swabs by tissue culture. (C) Ratio of HSV-2 strains in the cervicovaginal secretions. PCR was used to differentiate the strains of HSV-2 detected in secretions. HSV-2 strain 333 levels were used as a baseline (white bars, ratio of 333 to 333 = 1), the ratio of HSV-2 186 levels to HSV-2 333 levels is shown by gray bars.

Four mature female RM (group 1) were inoculated intravaginally with 1 ml of a 1:1 mixture of 2 HSV-2 strains (strains 186 and 333; total titer of 10^7^ PFU) on days 0, 7, 14, 21, and 56 (Fig. 1). As we are unsure if there was a difference in the abilities of different HSV-2 strains to infect RM, we used a mixture of HSV-2 strains for the inoculations. Infectious virus and HSV-2 DNA were consistently detected in secretions of all 4 animals for the first 7 days after every inoculation (Fig. 1A and B). HSV-2 DNA was detected in all vaginal swabs taken within 7 days of the initial intravaginal inoculation (Fig. 1A), and replication-competent HSV-2 was isolated in tissue culture on 30 of the 78 (38%) samples submitted for virus isolation during the same period (Fig. 1B). As expected, HSV-2 DNA was detected in all genital secretion samples that were viral isolation positive. HSV-2 DNA detection decreased nearly linearly (10^7^ to 10^2^ copies/swab) over the first 10 to 14 days postinoculation. The titer and duration of HSV-2 DNA shedding in secretions were similar after each of the initial 4 weekly HSV-2 inoculations. Clinically, no genital lesions, fever, or change in appetite, behavior, or bowel or motor functions were noted postinoculation. Importantly, spontaneous subclinical shedding of HSV-2 DNA (10^2^ to 10^3^ copies/swab) was detected in secretions collected between day 42 (outside the acute phase of intense virus shedding) and day 56 (Fig. 1A) in 3 of the 4 animals. HSV-2 DNA was intermittently shed in the secretions of these 3 animals during this period. The duration of each shedding episode was less than 1 day, meaning that DNA was detected in only one of the two swabs collected 8 h apart on that day (Fig. 1A) and was at titers of 10^2^ to 10^3^, as seen with subclinical shedding in humans (14, 16). The animals were necropsied 7 to 17 days after the last HSV-2 inoculation on day 56.

A real-time PCR assay that distinguishes between HSV-2 strains 186 and 333 was used to identify the viruses present in genital secretions during the acute phase of high-level HSV-2 DNA shedding (Fig. 1C). In almost all samples tested, both strains were detected; HSV-2 strain 186 was somewhat more common, especially at later time points.

**FIG 2:**
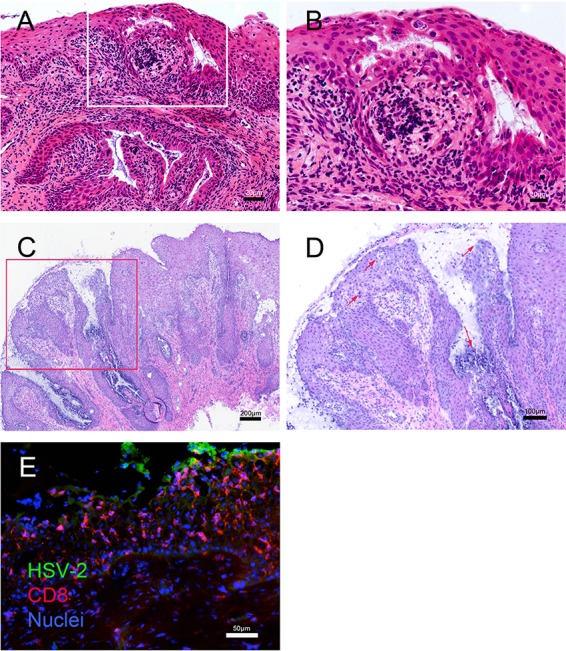
Histopathology and localization of HSV-2 antigen and CD8^+^ T cells in cervical biopsy tissue obtained 2 and 7 days after vaginal HSV-2 inoculation. Panels A and B show formalin-fixed paraffin-embedded sections of a cervical biopsy specimen collected at 2 days p.i. (A) H&E stain of ectocervix. The overlying epithelium is intact but is compromised by microvesicles and clefts filled with fibrin and cellular debris. Inflammatory cell infiltrates surround small blood vessels in the lamina propria and extend into the stratified epithelium of the ectocervix. Lymphatic vessels in the lamina propria are distended with inflammatory cells and edema fluid. (B) Higher magnification of the inset from panel A. The inflammatory cell infiltrate is focused on the epithelium and is comprised mainly of mononuclear cells, with fewer neutrophils. There is widespread acantholysis in the stratified squamous epithelium, with ballooning degeneration of epithelial cells. The microvesicles within the epithelium contain fibrin, cell debris, acantholytic epithelial cells, and inflammatory cells. Many of the epithelial cells at the edge of the microvesicles have large ground-glass nuclei with marginated chromatin. Panels C, D, and E show frozen sections of a cervical biopsy specimen collected at 7 days p.i. (C) H&E stain of the transformation zone at the border of the endocervix and ectocervix. Much of the overlying epithelium is lost or degenerating, replaced by fibrin, inflammatory cells, and cellular debris. The remaining epithelium contains acellular areas filled with fibrin, cellular debris, and edema. Inflammatory cell infiltrates surround small blood vessels in the lamina propria and extend into the columnar epithelium of the endocervix and the stratified squamous epithelium of the ectocervix. Lymphatic vessels in the lamina propria are distended with inflammatory cells and fluid. (D) Higher magnification of the inset from panel C. The inflammatory cell infiltrate is focused on the epithelium and is comprised mainly of mononuclear cells, with fewer neutrophils. There is widespread acantholysis in the stratified squamous epithelium, with ballooning degeneration of epithelial cells. The arrows indicate microvesicles within the epithelium that contain fibrin, cell debris, and inflammatory cells. (E) Immunofluorescent staining for HSV-2 antigen (green) and CD8^+^ T cells (red). Note that the HSV-2 antigen is expressed in the middle layer of the epithelium by acantholytic or detached epithelial cells at the edge of intraepithelial vesicles.

**FIG 3:**
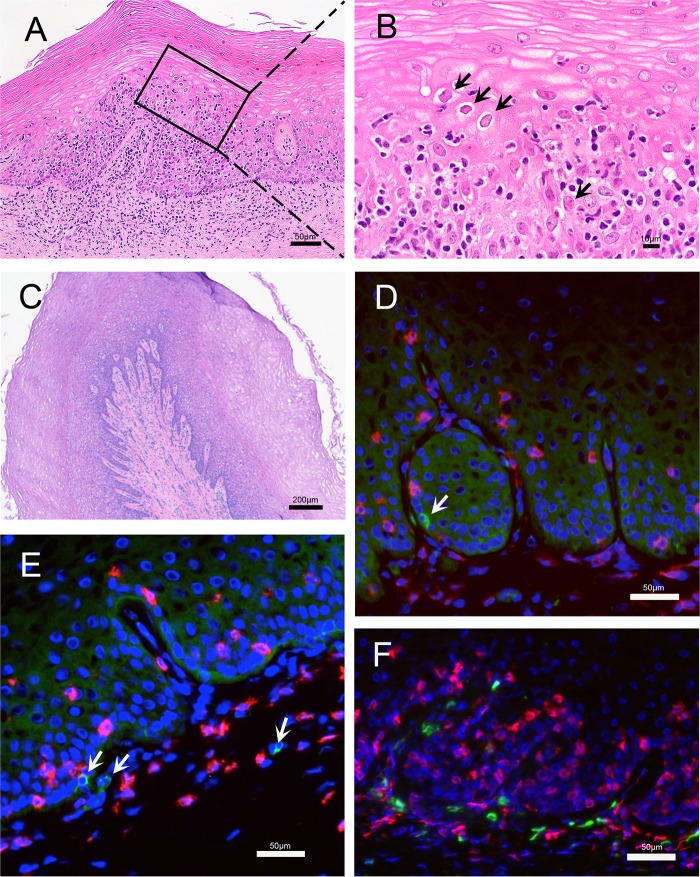
Histopathology and localization of HSV-2 antigen and T cells in vaginal mucosa obtained at necropsy. (A and B) Images are from a region of vaginal mucosa with obvious histopathology. (A) Within the squamous epithelium and lamina propria there were multiple focal areas with mononuclear inflammatory cell infiltrates. In the lamina propria, the inflammatory cells surrounded small blood and lymphatic vessels that were distended by edema. In the stratified squamous epithelium, the inflammatory cell infiltrates were focused in areas with acantholytic and degenerating epithelial cells. (B) Higher magnification of the inset from panel A. There is widespread acantholysis in the stratified squamous epithelium with ballooning degeneration of epithelial cells. The arrows indicate some of the numerous eosinophilic (red) intranuclear inclusion bodies and marginated chromatin within epithelial cells that are characteristic of herpesvirus infection. (C to F) Images are from a region of the vaginal mucosa with mild histopathology. (C) Normal epithelium with numerous layers characteristic of the follicular phase of the menstrual cycle. There is a mild mononuclear cell infiltrate with edema centered on the basal epithelial layer, superficial lamina propria, and around small vessels. Lymphatic vessels are distended with fluid. (D and E) Immunofluorescent staining for HSV-2 antigen (green) and CD8^+^ T cells (red). HSV-2 antigen is expressed in a few basal epithelial cells and mononuclear cells in the lamina propria (arrows). (F) Immunofluorescent staining for CD4^+^ (green) and CD8^+^ (red) T cells.

Histologic evidence of active HSV-2 infection in the genial tract tissue was found in genital tissue collected by biopsy at day 7 postinoculation (p.i.) and at necropsy 7 to 17 days after the last HSV-2 inoculation (Fig. 2 and 3). In the cervical biopsy specimens from all 4 animals collected at 7 days p.i. there were multifocal areas where the overlying epithelium was lost or degenerating and widespread acantholysis and degeneration of epithelial cells (Fig. 2A and B). In some areas the upper layers of the epithelium were lost, and in other areas clefts were forming between epithelial layers (Fig. 2C and D). These are both changes consistent with different stages in the formation and rupture of fluid-filled vesicles. The cytopathic changes in the epithelium were associated with an intense inflammatory cell infiltrate, comprised mainly of mononuclear cells with fewer neutrophils, surrounding small blood vessels in the lamina propria and extending into the columnar epithelium of the endocervix and the stratified squamous epithelium of the ectocervix. Lymphatic vessels in the lamina propria were distended with inflammatory cells and fluid (Fig. 2A and B). Importantly, HSV-2 antigen was detected in the epithelium in both the vagina and cervix (Fig. 2E). Areas with HSV-2 antigen had a dense CD8^+^ T-cell infiltrate (Fig. 2E). A complete examination of the genital tract at necropsy 7 to 17 days after the last HSV-2 inoculation on day 56 revealed no evidence of herpetic ulcers. However, on histologic examination, all 4 animals had a chronic cervicitis and vaginitis characterized by multiple, variably spaced, focal areas of inflammation (Fig. 3A). The cellular infiltrate associated with the inflammation was largely mononuclear and was focused on areas of epithelial cell degeneration and acantholysis. In addition, epithelial cells with the eosinophilic (red) intranuclear inclusion bodies and marginated chromatin, which are characteristic cytopathic effects of herpesvirus infections, were common in these areas (Fig. 3B). Even in areas of the vaginal mucosa with little inflammation (Fig. 3C), HSV-2 antigen was found in a few basal epithelial cells and, rarely, in mononuclear cells in the lamina propria (Fig. 3D and E). Both CD4^+^ and CD8^+^ T cells were numerous in the vaginal mucosa of these chronically HSV-2-infected animals (Fig. 3F). Cervical biopsy specimens collected at day 7 after the initial inoculation in the 4 animals in group 1 revealed HSV DNA in tissue at high copy numbers (1,100 to 5,400 copies/million cells), while none of the cervical biopsy specimens taken at day 28 from the 6 RM in group 2 were HSV-2 DNA positive (Table 1). Thus, evidence of genital tissue infection, viral replication, and an associated inflammatory response was seen histologically at 7 days p.i.

**TABLE 1:** HSV-2 DNA in tissues of HSV-2-inoculated rhesus macaques

Tissue	No. of copies of HSV-2 DNA/million cells in indicated animal												
	Group 1 (live virus inoculum)				Group 2 (live virus inoculum)						Group 5 (killed virus inoculum)		
	34806	34889	35667	38420	33869	35633	37957	34099	39406	33991	40257	38044	37349
Cervical biopsy^*a*^	1,147	2,184	5,448	3,271	0	0	0	0	0	0	346	0	68
Vulva^*b*^	0	0	38	3,270	20,543	3,871	434	2,901	185	185	0	0	0
Cervix^*b*^	0	51	144	0	204	581	936	51	128	0	0	0	0
Vagina 1^*b*^^,^^*c*^	3,376	155	0	608	16,855	1,004	863	133	63	76	0	0	0
Vagina 2^*b*^^,^^*d*^	1,803	188	0	212	3,745	4,400	1,208	252	133	0	0	0	0
Vagina 3^*b*^^,^^*e*^	0	0	0	0	332	1,283	1,449	293	117	642	0	0	0
Cervical ganglia/cord^*b*^	0	7,719	0	915	0	147	0	64	0	0	0	0	0
Thoracic ganglia/cord^*b*^	0	0	0	6,946	0	0	0	0	0	0	0	0	0
Lumbar ganglia/cord^*b*^	0	0	0	0	425	0	0	3,043	68	0	0	0	0
Sacral ganglia/cord^*b*^	0	0	0	0	0	0	0	0	0	0	0	0	0
Pelvic nerve	656	0	0	0	0	0	0	0	0	0	0	0	0
Sacral systemic trunk	ND^*f*^	ND	ND	ND	1,590	0	0	0	0	0	0	0	0

a Biopsy specimens were collected 7 days after the first HSV-2 inoculation for group 1, 28 days after the first HSV-2 inoculation for group 2, and 2 or 3 days after the first heat-killed HSV-2 inoculation for group 5.

b Necropsy tissue collected 2 to 7 days after the last HSV-2 inoculation.

c Proximal vaginal mucosa (fornix).

d Mid-vaginal mucosa.

e Distal vaginal mucosa (introitus).

f ND, not determined.

**FIG 4:**
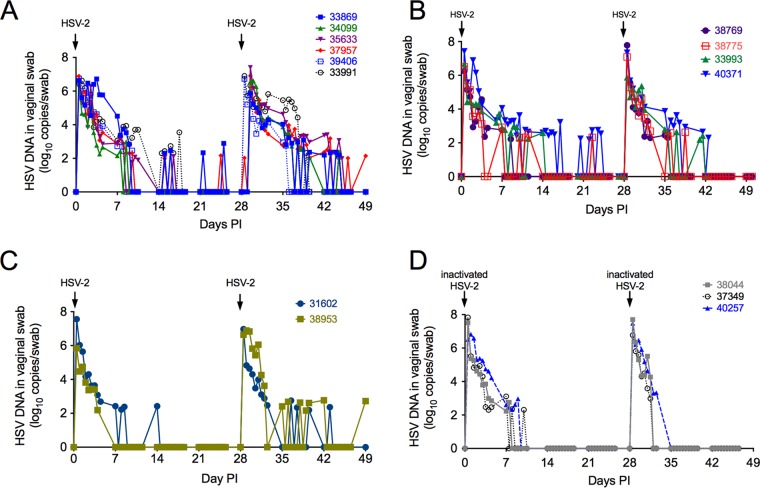
Spontaneous reactivation of HSV-2 in RM is detected after infectious viral challenge but not after heat-inactivated HSV-2 challenge. (A) Four of the six female monkeys in group 2 were intravaginally inoculated with a mixture of HSV-2 strains 186 and 333 (total of 1 × 10^7^ PFU in the 1-ml inoculum) (solid lines and solid symbols); the remaining 2 animals were inoculated with a mixture of HSV-2 strains 186, 333, and G (dashed lines and open symbols). Inoculations were separated by 28 days. (B) Group 3. These 4 animals were treated with medroxyprogesterone acetate and then inoculated 2 times with HSV-2 (days 0 and 28). (C) Group 4. These 2 animals were treated with a depleting antibody that targets CD8α^+^ lymphocytes (T cells and NK cells) and then inoculated 2 times with HSV-2 (days 0 and 28). (D) Group 5. These three female monkeys were intravaginally inoculated with a mixture of heat-killed HSV-2 strains 186, 333, and G (1 × 10^7^ PFU of HSV-2/ml prior to inactivation). Note that 11 of 12 RM inoculated with live HSV-2, but none of the 3 animals inoculated with heat-killed HSV-2, shed HSV-2 DNA in genital secretions at least once between days 14 to 28 and 35 to 49 postinoculation.

To evaluate whether subclinical reactivation occurs in RM, we intravaginally inoculated 6 additional animals (group 2) with HSV-2 twice, on day 0 and day 28 (Fig. 4A). Four of the 6 RM were inoculated intravaginally with 1 ml of a 1:1 mixture of 2 HSV-2 strains (186 and 333; total titer of 10^7^ PFU), and 2 of the 6 RM were inoculated with 1 ml of a 1:1:1 mixture of 3 HSV-2 strains (G, 186, and 333; total titer of 10^7^ PFU) (Fig. 4A). As before, HSV-2 DNA was consistently detected in secretions of all 6 animals for approximately the first 10 days after every inoculation (Fig. 4A). Importantly, spontaneous HSV-2 reactivation as detected by HSV-2 DNA (10^2^ to 10^3^ copies/swab) in swabs occurred in 5 of the 6 animals during the 14- to 28- and 35- to 100-day postinoculation periods (Fig. 4A). The duration of shedding episodes ranged from less than 1 day to 2 days (Fig. 4A), a pattern of intermittent subclinical shedding that is similar to that seen in women with HSV-2 (19, 20). However, the frequency of shedding decreased as the interval after inoculation increased, and the last DNA-positive samples were collected at 60 days p.i., 32 days after the final HSV-2 inoculation (Fig. 4A).

### Effect of medroxyprogesterone acetate administration on HSV-2 infection of RM.

Pretreatment with exogenous progestins, such as medroxyprogesterone acetate (Depo-Provera), has been reported to facilitate vaginal HSV-2 infection of mice and SIV/SHIV infection in RM because it increases epithelial permeability and thins the stratified squamous epithelium by removing the cornified layers (21). To determine if medroxyprogesterone acetate markedly altered HSV-2 infection in RM, medroxyprogesterone acetate (30 mg/animal) was administered to 4 animals (group 3) by intramuscular injection 30 days prior to, and on the day of, the first intravaginal inoculation with 1 ml of a 1:1:1 mixture of 3 HSV-2 strains (G, 186, and 333; total titer of 10^7^ PFU). The animals were challenged with HSV-2 twice, separated by 28 days (Fig. 4B). The pattern of HSV-2 DNA shedding was similar to that of the no-medroxyprogesterone acetate group 2 animals; viral DNA (vDNA) was consistently detected in secretions of all 4 animals for 7 to 14 days after every inoculation (Fig. 4B). As with the no-medroxyprogesterone acetate group 2 animals, spontaneous HSV-2 DNA (10^2^ to 10^3^ copies/swab) was documented in 3 of 4 medroxyprogesterone acetate-treated animals during the 14- to 28- and 35- to 100-day postinoculation periods. During these periods, the duration of shedding was never more than 24 h (Fig. 4B) and the frequency of shedding decreased over time, with the last HSV-2 DNA samples collected at 60 days p.i. (32 days after the final HSV-2 inoculation). Thus, although our sample size was small, medroxyprogesterone acetate did not markedly alter the pattern of acute HSV-2 shedding or subsequent HSV-2 reactivation in RM.

### Effect of transient CD8α chain-positive lymphocyte (T cell and NK cell) depletion on HSV-2 infection of RM.

NK cells can recognize decreased surface expression of major histocompatibility complex class I (MHC-I) on virus-infected cells immediately after infection, and kill these cells, before adaptive immune responses have developed. To determine the effect of naive CD8^+^ T cells and NK cells on HSV-2 infection in RM, a anti-CD8α chain-depleting monoclonal antibody (MAb) and an interleukin 15 (IL-15)-depleting MAb were administered by intravenous (i.v.) injection to 2 animals (group 4) by subcutaneous injection 3 days prior to, and on the day of, intravaginal inoculation with 1 ml of a 1:1:1 mixture of 3 HSV-2 strains (G, 186, and 333; total titer of 10^7^ PFU). While all CD8α^+^ cells (T cells and NK cells) in blood were completely depleted (data not shown), we and others have previously shown that approximately 20% of CD8α^+^ cells in mucosal tissues persist after anti-CD8α MAb administration (22). The animals were challenged with HSV-2 twice, separated by 28 days (Fig. 4C). The pattern of HSV-2 DNA shedding was similar to that in the untreated group 2 animals and the medroxyprogesterone acetate-treated group 3 animals; vDNA was consistently detected in secretions from both animals for 7 to 14 days after every inoculation (Fig. 4C). Spontaneous HSV-2 DNA shedding (10^2^ to 10^3^ copies/swab) that persisted for less than 24 h was detected in the animals during the 14- to 28- and 35- to 100-day postinoculation periods, and the last HSV-2 DNA-positive cervicovaginal secretion (CVS) sample was collected 64 days p.i. (36 days after the final HSV-2 inoculation) (Fig. 4C). These data suggest no dramatic difference in HSV-2 shedding with this form of CD8^+^ T-cell depletion.

### Shedding of HSV-2 DNA after vaginal inoculation of RM with heat-inactivated virus.

Three animals (group 5) were intravaginally inoculated twice (days 0 and 28) with 1 ml of a 1:1:1 mixture of 3 heat-inactivated HSV-2 strains (G, 186, and HSV-2 333; total titer of 10^7^ PFU) (Fig. 4D). None of the samples collected for viral isolation between days 1 and 3 had HSV isolated in tissue culture. However, HSV-2 DNA was detected in swab samples in the first week after inoculation with heat-inactivated virus. Importantly, histologic examination of the tissues revealed no evidence of cytopathology in the vulva and cervix in the heat-inactivated virus-inoculated animals, and spontaneous HSV-2 reactivation as determined by vDNA shedding was never detected during the 14- to 28- and 35- to 100-day postinoculation periods in these 3 animals (Fig. 4D). As discussed below, no evidence of neuronal HSV infection was seen at necropsy of animals inoculated with heat-killed HSV-2 (Table 1).

To compare the levels of HSV-2 DNA shedding after vaginal inoculation of RM with heat-inactivated virus and live HSV-2, the area under the curve (AUC) and decay rate of the HSV-2 DNA level in secretions for each animal over time intervals specified in “Statistical methods” below were calculated. The AUCs and decay rates of 2 groups were compared using the two-sample *t* test and Wilcoxon rank sum test (WRS). As medroxyprogesterone acetate treatment had no effect of HSV-2 DNA shedding in RM (statistical data not shown), these 2 animal groups were combined in a subsequent analysis to compare HSV-2 DNA shedding in animals inoculated with live HSV-2 (groups 2 and 3) versus animals inoculated with heat-inactivated HSV-2 (group 5).

Significantly less HSV-2 DNA was shed in group 5 than in groups 2 and 3 from day 0.5 to 39.5 p.i. as determined by comparison of AUCs by the *t* test (*P* = 0.04) but not the WRS (*P* = 0.112), and the decay rate of HSV DNA level was significantly higher in the inactivated HSV animals (group 5) than in live HSV-2 animals (groups 2 and 3) after the second inoculation (days 28.5 to 39.5 p.i.) by both the *t* test (*P* = 0.003) and the WRS (*P* = 0.007). Thus, the results of these statistical analysis support the conclusion that while much of the HSV-2 DNA detected in the first 12 days after inoculation is due to decay of the virus in the inoculum, productive viral replication also contributes to viral shedding in this phase. Further productive viral replication accounts for all the HSV-2 DNA shedding outside the initial postinoculation period of intense viral shedding.

### Proteomic analysis of CVS after vaginal inoculation of RM with live HSV-2 or heat-inactivated virus.

**FIG 5:**
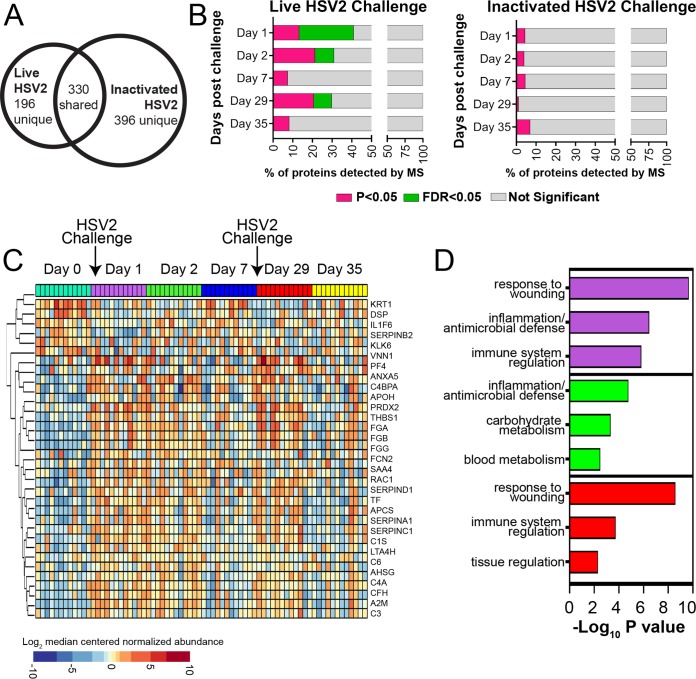
Mucosal wound healing and inflammatory responses were observed in CVS after live HSV-2 inoculation. (A) Using mass spectrometry, a total of 526 and 726 proteins were detected in CVS samples from live HSV-2-challenged RM (groups 2, 3, and 4; *n* = 12) and inactivated HSV-2-challenged macaques (group 5; *n* = 3), respectively. (B) Significantly differentially expressed proteins were identified on days 1 and 2 after live HSV-2 challenge relative to baseline (day 0). The proportion of proteins that were significantly different at an unadjusted *P* value of <0.05 (pink) and adjusted *P* value of <0.05 (FDR = 5% [green]) are displayed. Significant protein changes were also observed 1 day after a second live HSV-2 challenge (day 29). No significant protein alterations were observed after challenge with inactivated HSV-2 (*P* > 0.05). (C) Differentially expressed proteins in CVS of 12 RM (groups 2, 3, and 4) after live HSV-2 challenge displayed as a heat map. (D) The top 3 functions associated with proteins that were significantly altered in groups 2, 3, and 4 on days 1 (purple bars), 2 (green bars), and 29 (red bars) by live HSV-2 challenge were related to wound healing, innate immunity, and inflammation.

CVS samples collected from RM in groups 2, 3, 4, and 5 were analyzed by mass spectrometry to identify protein signatures that were uniquely associated with live HSV-2 replication within the vaginal mucosa. A total of 526 proteins were detected across all time points from live HSV-2-challenged RM (groups 2, 3, and 4) samples, while 726 proteins were detected from inactivated HSV-2-challenged RM (group 5) samples (Fig. 5A). Protein expression was evaluated against baseline and the prechallenge time point within each study group. Proteome alterations were observed on days 1, 2, and 29 in CVS from RM inoculated with live HSV-2 (groups 2, 3, and 4), which were significant after multiple hypotheses testing correction. However, by 7 days p.i. (days 7 and 35), the proteome was not significantly different than the day 0 proteome (Fig. 5B). Critically, no significant changes were observed after inactivated HSV-2 challenge (group 5) at any time point (Fig. 5B). Functional enrichment analysis was performed on proteins differentially expressed after live HSV-2 challenge using the Gene Ontology database; this analysis showed that inflammation and wound-healing processes were significantly associated with these proteins. Visualization by hierarchical clustering showed a clear increase in these inflammatory proteins at days 1 and 2 after live HSV-2 exposure (Fig. 5C). The top three unique functions enriched on days 1, 2, and 29 are displayed (Fig. 5D). Proteins involved in wounding responses, inflammation, and antimicrobial defense were enriched at all three of these time points postchallenge (*P* < 0.0001) (Table 2).

**TABLE 2:** Functional enrichment analysis of CVS proteome in live HSV-2-inoculated RM

Function type and term	Count	*P* value	Bonferroni	Genes
Functions associated with proteins differentially expressed on day 1 relative to baseline (day 0)				
Response to wounding	31	1.51E−13	6.97E−11	A2M, C3, C6, C5, PRDX2, PF4, AHSG, FGG, FGA, FGB, FCN2, RAC1, SERPINC1, CFH, KRT1, APOH, VNN1, SERPINA1, THBS1, KLK6, APCS, C4A, SAA4, C4BPA, ANXA5, SERPINB2, DSP, LTA4H, SERPIND1, IL1F6, PROS1
Defense response	24	3.50E−07	1.62E−04	A2M, APCS, C4A, C3, S100A7, C6, C5, SAA4, PRDX2, CD5L, HP, C4BPA, COTL1, AHSG, FCN2, RAC1, KRT1, CFH, LTF, VNN1, LTA4H, SERPINA1, THBS1, IL1F6
Regulation of immune system process	18	1.48E−06	6.84E−04	A2M, C4A, C3, IL6ST, C6, C5, PF4, PRDX2, C4BPA, AMBP, APOA1, HPX, FCN2, CFH, KRT1, VNN1, CA2, THBS1
Blood coagulation	10	2.09E−06	9.65E−04	FGG, FGA, FGB, APOH, SERPINC1, PF4, SERPINA1, SERPIND1, ANXA5, PROS1
Humoral immune response	9	2.77E−06	1.28E−03	C4A, C3, FCN2, C6, C5, CFH, KRT1, LTF, C4BPA
Regulation of immune response	13	8.99E−06	4.16E−03	AMBP, A2M, APOA1, C4A, HPX, IL6ST, C3, FCN2, C6, C5, KRT1, CFH, C4BPA
Carbohydrate catabolic process	9	3.04E−05	1.40E−02	GNS, TPI1, GM2A, PGD, PGAM1, BPGM, GAPDH, MDH1, ENO1

Functions associated with proteins differentially expressed on day 2 relative to baseline (day 0)				
Inflammatory response	17	1.12E−08	7.48E−06	TF, A2M, APCS, C4A, C3, C6, C5, SAA4, PRDX2, C1S, AHSG, FCN2, KRT1, CFH, VNN1, SERPINA1, THBS1
Carbohydrate catabolic process	10	3.01E−07	2.02E−04	TPI1, GM2A, NUDT5, PGD, MGAM, PGAM1, BPGM, GAPDH, MDH1, ENO1
Blood coagulation	9	2.09E−06	1.40E−03	FGG, FGA, FGB, APOH, SERPINC1, SERPINA1, SERPIND1, ANXA5, PROS1
Regulation of immune response	12	2.85E−06	1.91E−03	AMBP, A2M, APOA1, C4A, C3, HPX, FCN2, C6, C5, CFH, KRT1, C1S
Epidermis development	11	6.24E−06	4.17E−03	KRT6A, KRT17, KRT5, KRT16, KRT15, KRT1, ALOX12B, DSP, KRT13, KRT2, KRT10
Response to metal ion	9	1.21E−05	8.05E−03	ACTB, FGG, APOB, FGA, FGB, ANXA11, SERPINA1, CA2, THBS1
Wound healing	10	3.09E−05	2.05E−02	FGG, FGA, FGB, APOH, SERPINC1, DSP, SERPINA1, SERPIND1, ANXA5, PROS1

Functions associated with proteins differentially expressed on day 29 relative to baseline (day 0)				
Response to wounding	25	6.32E−12	2.60E−09	TF, A2M, C3, C5, PF4, PRDX2, C1S, AHSG, FGG, FGA, FGB, KRT1, SERPINC1, VNN1, SERPINA1, THBS1, APCS, C4A, C4BPA, ANXA5, VWF, DSP, SERPIND1, IL1F6, PROS1
Blood coagulation	10	1.53E−07	6.30E−05	VWF, FGG, FGA, FGB, SERPINC1, PF4, SERPINA1, SERPIND1, ANXA5, PROS1
Defense response	19	2.62E−06	1.07E−03	TF, A2M, APCS, C4A, C3, S100A7, C5, CD5L, HP, PRDX2, C4BPA, C1S, COTL1, AHSG, KRT1, VNN1, SERPINA1, THBS1, IL1F6
Regulation of immune system process	14	1.66E−05	6.80E−03	A2M, APOA1, C4A, HPX, C3, C5, KRT1, VNN1, PRDX2, PF4, CA2, C1S, C4BPA, THBS1
Complement activation	6	2.32E−05	9.50E−03	C4A, C3, C5, KRT1, C1S, C4BPA
Tissue development	17	1.07E−04	4.30E−02	MYL6, KRT6A, S100A7, KRT13, KRT10, SCEL, FGA, KRT16, KRT15, AGT, KRT1, ALOX12B, DSP, KRT2, KRT3, KRT4, CA2

### Systemic HSV-2-specific T-cell responses.

**FIG 6:**
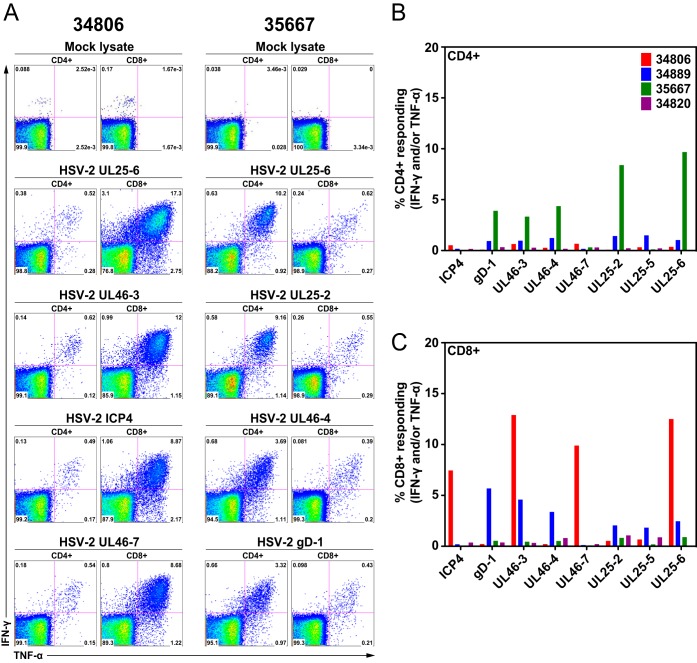
HSV-2-specific CD4^+^ and CD8^+^ T-cell responses in splenocytes of four HSV-2-infected rhesus macaques (group 1). Splenocytes of four monkeys were stimulated with various HSV-2 peptide pools. HSV-2-specific, TNF-α- and IFN-γ-secreting CD4^+^ and CD8^+^ T cells were detected by flow cytometric intracellular cytokine staining. (A) Representative data from 2 animals (35667 and 34806) stimulated with various HSV-2 peptide pools or a mock cellular lysate as indicated above each dot plot. For each animal, CD4^+^ T-cell responses are on the left and CD8^+^ T-cell responses are on the right. (B) Frequency of CD4^+^ T cells producing TNF-α and IFN-γ in response to stimulation with the peptide pools indicated on the *x* axis. (C) Frequency of CD8^+^ T cells producing TNF-α and IFN-γ in response to stimulation with the peptide pools indicated on the *x* axis.

To determine if the group 1 animals (inoculated 5 times) made HSV-2-specific T-cell responses, cryopreserved splenocytes were stimulated with HSV-2 peptides pools or a lysate of mock-infected cells. HSV-2-specific, tumor necrosis factor alpha (TNF-α)- and gamma interferon (IFN-γ)-secreting CD4^+^ and CD8^+^ T cells were detected by flow cytometric intracellular cytokine staining (Fig. 6). All 4 animals made easily detectable T-cell responses to a wide variety of HSV-2 antigens, including the capsid/tegument and surface glycoproteins. Two of the four animals made very strong anti-HSV-2 responses (>5% of a T-cell subset), but one these animals (35667) had a response dominated by CD4^+^ T cells, and in the other animal (34806) the response was made largely by CD8^+^ T cells (Fig. 6).

**FIG 7:**
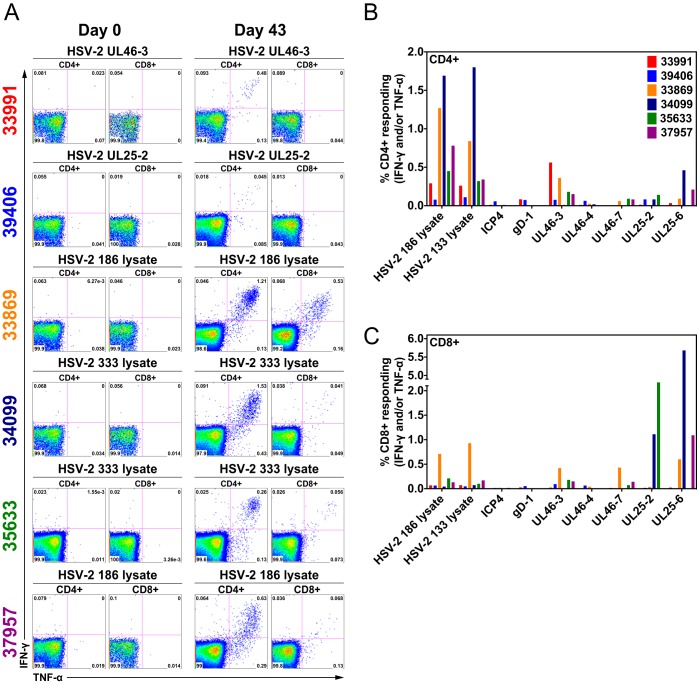
HSV-2-specific CD4^+^ and CD8^+^ T-cell responses in PBMC from six HSV-2-infected rhesus macaques (group 2). PBMC collected prior to HSV-2 inoculation and 43 days after the first HSV-2 inoculation were stimulated with a lysate of HSV-2-infected cells or various HSV-2 peptide pools. HSV-2-specific, TNF-α- and IFN-γ-secreting CD4^+^ and CD8^+^ T cells were detected by flow cytometric intracellular cytokine staining. (A) Representative data from 6 animals stimulated with various HSV-2 peptide pools or HSV-2 lysates as indicated above each dot plot. For each time point, CD4^+^ T-cell responses are on the left and CD8^+^ T-cell responses are on the right. (B) Frequency of CD4^+^ T cells in day 63 PBMC producing TNF-α and IFN-γ in response to stimulation with the HSV-2 lysates and HSV-2 peptide pools indicated on the *x* axis. (C) Frequency of CD8^+^ T cells in day 63 PBMC producing TNF-α and IFN-γ in response to stimulation with the HSV-2 lysates and HSV-2 peptide pools indicated on the *x* axis.

To determine if the group 2 animals made HSV-2-specific T-cell responses, aliquots of cryopreserved peripheral blood mononuclear cells (PBMC) collected before HSV-2 inoculation (day 0) and at day 43 p.i. were stimulated with HSV-2 peptide pools, lysates of HSV-2-infected cells, and a lysate of mock-infected cells. HSV-2-specific, TNF-α- and IFN-γ-secreting CD4^+^ and CD8^+^ T cells were detected by flow cytometry and intracellular cytokine staining (Fig. 7). All 6 animals made readily detectable T-cell responses to HSV-2 antigens, but there was wide variation in the strength and breadth of the responses (Fig. 7). One animal (39406) made very weak CD4^+^ and CD8^+^ T-cell responses to all HSV-2 antigens tested, and 2 animals (34099 and 33869) had relatively strong HSV-2-specific T-cell responses (Fig. 7). As expected, HSV-2-specific CD4^+^ T-cell responses were strongest and most consistent in PBMC stimulated with the HSV-2-infected cell lysates, but peptide pools elicited the strongest HSV-2-specific CD8^+^ T-cell responses (Fig. 7).

### HSV-2-specific antibody responses.

Acute- and convalescent-phase sera collected from days 42 to 350 p.i. were analyzed. Western blotting and enzyme-linked immunosorbent assays (ELISAs) using whole HSV-2 antigen did not detect HSV-2-specific IgG in plasma of the 4 inoculated animals that were tested (see Table S3 in the supplemental material). IgG antibodies to glycoprotein G (gG) of HSV-2 were detected in 2 animals (35633 and 40371) using the HerpeSelect ELISA, while a Luminex-based assay detected IgG antibodies to gD2 of HSV-2 in 3 animals (35633, 39406, and 34889) (Table S3). One of these animals (35633) also demonstrated rising titers to the capsid protein UL-19. This low frequency of seroconversion to HSV-2 among the infected RM was unexpected but may reflect the relatively low levels of HSV-2 replication in the RM.

### HSV-2 DNA in neural tissue.

Lastly, we tested for the presence of HSV-2 DNA and HSV-2 RNA in tissues (vulva, vagina, cervix, pelvic nerves, and sacral, lumbar, thoracic, and cervical ganglia) collected at necropsy from the group 1, group 2, and group 5 animals, as well as cervical biopsy specimens collected at 7 days after the first HSV-2 inoculation from group 1 and group 2 animals. Tissue was homogenized, and DNA was extracted and subjected to quantitative PCR (qPCR) for HSV-2 DNA (Table 1). Group 1 animals were necropsied at day 72, 14 to 18 days after the last HSV-2 inoculation, and group 2 animals were necropsied at days 50 to 57, 3 weeks after the last inoculation. All 3 group 5 animals receiving heat-inactivated HSV-2 were necropsied between days 161 and 211 p.i. As shown in Table 1, although we detected HSV DNA in sacral and/or autonomic ganglia (pelvic nerve) or sacral sympathetic trunk in 7 of the 10 animals that received live virus challenges; HSV DNA was not detected in neural tissue at necropsy in any of the animals receiving heat-inactivated virus challenge. Variability in titer was seen between sites and animals (Table 1).

HSV DNA was present in cervical tissue at day 7 in 4 of 10 group 1 and group 2 animals at necropsy, indicating cervical tissue infection (Table 1), even at times in which superficial cervical swabs were negative. HSV-2 DNA was not detected in any of the 18 vaginal or cervical tissues collected at necropsy from group 5 animals (Table 1). HSV mRNA for the latency-associated transcript (LAT) was not detected in the nervous tissue samples of any RM using quantitative reverse transcription-PCR (RT-PCR).

## DISCUSSION

Our data demonstrate acute HSV-2 infection, neural infection/persistence, and spontaneous and subclinical reactivation in RM for about 40 days after virus inoculation. We found that after vaginal inoculation with HSV-2, RM develop a subclinical infection with persistence of vDNA in the genital tract and nervous tissues, histologic evidence of viral pathology in the genital tract in the first few weeks after inoculation, transient increased expression of proteins associated with wound healing, innate immunity, and inflammation, and spontaneous intermittent low-level virus shedding in genital secretions up to 30 days after virus inoculation. Despite evidence of persistent ganglionic infection on autopsy, the frequency of HSV-2 shedding seemed to decrease as the interval between inoculation and sampling increased, suggesting that either HSV-2 reactivation is less frequent and spread more restricted or viral clearance is enhanced in RM compared to humans, in whom reactivation is quite frequent in the early time periods post primary acquisition (23).

Because the stratified squamous epithelia of the perineum, vagina, and ectocervix are the major sites of HSV-2 replication, determining the levels of virus shed in genital swabs collected at regular intervals is the standard method used to characterize the virology of HSV-2 infection in women. By comparing vDNA levels in secretions of groups of RM inoculated with aliquots of either active or inactivated HSV-2, we are able to conclude that while much of the HSV-2 DNA detected in the first 7 days after inoculation is due to decay of the virus inoculum, some shedding of DNA is associated with productive viral replication. Further, productive viral replication accounts for all the HSV-2 DNA shedding outside the initial postinoculation period. Although the animals were inoculated with a mixture of at least HSV-2 strains, there was not a detectable difference in the ability of any strain to infect RM, suggesting that none of these HSV-2 strains has a replicative advantage in RM. Recent studies of women using genital secretions collected daily have shown that brief episodes of clinically silent HSV-2 shedding are common (24, 25). Thus, for approximately 40 days after virus inoculation, the pattern of virus shedding found in RM is similar to that found in HSV-2^+^ asymptomatic women.

Although we are unaware of any studies describing the CVS proteome in women with acute HSV-2 infection, the pattern of protein expression in CVS of RM after HSV-2 inoculation which included proteins involved in the response to epithelial damage, antimicrobial defense, and inflammation that were enriched from 1 to 7 days after live HSV-2 inoculation (Fig. 5) is consistent with acute HSV-2 infection. Of note, the most striking responses occurred at 1 and 2 days p.i., with a trend toward a return to baseline protein expression by 7 days p.i. This pattern is consistent with an acute viral infection of the female reproductive tract with epithelial damage followed by repair and an ongoing immune and inflammatory response. These results confirm that the RM became infected with HSV-2, as changes in the CVS proteome were not seen in RM inoculated with killed HSV-2. The histopathology in cervical biopsy tissues and the genital tissues collected at necropsy was consistent with HSV-2 infection (subacute inflammatory infiltrates, intraepithelial vesicles and microulcers, and degenerating epithelial cells, including some with intranuclear inclusions) (26). HSV-2 DNA was consistently found in the same samples, confirming the presence of HSV-2 infection and related pathology in the genital tract of RM.

The detection of HSV antigen and DNA in tissue from inoculated RM strengthens the conclusion that the RM were infected by HSV-2. HSV-2 infects nervous system tissues in humans, especially spinal ganglia, where replication is generally limited, and clinical signs generally associated with viral infection of the nervous system are rare despite the lifelong persistence of the infection. Tissues from 10 animals were collected at necropsy, and HSV-2 DNA was detected in the peripheral nervous system in 7 of the 10 animals, including pelvic nerves and sympathetic trunk in 2 of the 10 (Table 1). While we detected HSV-2 DNA in these samples, we did not assay whether we could recover virus from neural tissue by cocultivation, nor could we demonstrate RNA transcription for LAT. Future studies will be needed to define the metabolic state of HSV DNA in neural tissue of RM.

All 16 animals inoculated with live HSV-2 became infected and exhibited intermittent viral shedding, indicative of *in vivo* replication. The level of vDNA shed tended to be lower in positive genital swabs than in humans, CVS samples collected more than 40 days p.i. were never HSV-2 DNA positive, and none of the animals had clinical signs of HSV-2 infection. These data suggest that although RM became infected with HSV-2 after vaginal inoculation, most animals supported only moderate levels of HSV-2 replication (5, 6) for a limited time. It has been demonstrated that TRIM5a expression in RM cells partially blocks HSV-2 replication *in vitro* (7), and this may contribute to *in vivo* resistance of RM to high-level HSV-2 replication and disease. Indeed, we also found that HSV-2 replicated poorly in LLK-MK2 cells (a rhesus macaque kidney cell line) compared to Vero cells (a vervet monkey kidney cell line that does not produce an antiviral interferon response) or human HeLa cells *in vitro* (data not shown).

The immune response to the infection was characterized by strong and consistent T-cell responses but weak and variable antibody responses. Unexpectedly, only 30% of the HSV-2-inoculated RM animals seroconverted during the study. This low frequency of seroconversion may be due to the low-level, transient HSV-2 replication in the RM that provides insufficient antigen to drive maturation of B-cell responses. Another explanation is that longer follow-up and maturation of the B-cell response are needed in RM. In humans, many HSV antibody responses evolve slowly over time (27). These potential explanations are consistent with reports that antiviral therapy reduces HSV-2 shedding and retards the development of antibody to a number of HSV antigens in women (28) and that T-cell responses to HSV are present in some HSV-2-seronegative humans with no detectable virus shedding (29). Of note, at assignment to the study, all RM were negative for herpesvirus simiae (herpes B virus), and they had a variety of MHC and TRIM5a haplotypes (data not shown). However, there was no evidence of clinical herpesvirus disease in any of the HSV-2-infected macaques, and we did not detect macroscopic ulcers in the genital tract or sustained periods of high-level virus shedding in the animals. The RM model we describe here has many characteristics that are found in HSV-2 infection of immunocompetent women: spontaneous subclinical genital herpes, including the highly variable frequency of virus shedding between animals, and sporadic reactivation in individual animals. Whether long-term reactivation takes place over extended periods of follow-up requires further study.

Neither transient CD8α^+^ lymphocyte (T cell and NK cell) depletion nor medroxyprogesterone acetate treatment of RM prior to HSV-2 inoculation enhanced virus replication or shedding. Although both of these experiments involved very small numbers of animals, it was apparent that there was not a dramatic effect on HSV-2 infection in any of the treated animals. Several caveats apply to the CD8α depletion experiments. The RM were HSV-2-naive animals that had not made HSV-2-specific T-cell responses, so NK cells that lyse virus-infected cells due to decreased MHC-I expression on the surface of infected cells were the only antiviral effector mechanism that could have been affected by the preinoculation depletion of CD8α^+^ T cells. Future studies will include depletion of CD8α^+^ cells from chronically infected animals to determine if the cells have a role in suppressing HSV-2 replication at later stages. However, this approach has its limitations because anti-CD8α treatment does not deplete tissue-resident T cells (22), which are likely the cells most involved in suppressing viral replication (8, 30).

Our results may explain why prior attempts to infect RM with HSV have largely failed (10, 11). Without the twice-daily CVS sample collection, use of heat-killed HSV-2 to inoculate control animals, CVS proteomics, and sensitive T-cell assays, we would have been unable to conclude that the RM in this study became infected with HSV-2. Consistent to some degree with the results reported here, Crostarosa et al. reported that RM became infected after experimental vaginal HSV-2 inoculation and made poor HSV-2 antibody responses (13). However, they also reported that months after HSV-2 inoculation, viral DNA was regularly detected in genital swabs collected over a few days (13), suggesting that virus shedding was more persistent and consistent than in the present study.

In summary, all 16 RM that were vaginally inoculated with HSV-2 developed acute HSV-2 infection evidenced by viral shedding in genital secretions, increased expression of proteins associated with tissue damage and inflammation in the genital tract, and tissue-based evidence of HSV-2 replication in the genital tract. Importantly, sporadic HSV-2 DNA shedding in genital secretions for 40 days p.i. provided evidence of transient reactivation of virus replication in 13 of 16 animals. The HSV-2^+^ animals developed an impressive T-cell response to a wide variety of viral antigens; some animals developed antibodies to capsid and surface glycoproteins, and we detected evidence of HSV-2 DNA in sensory and autonomic ganglia. The intense multifocal inflammatory response in the genital tract during both acute and chronic infection provides optimism that the model may be a useful one in dissecting the mechanisms underlying immune control of HSV-2 in mucosal surfaces. The presence of viral antigen and immune cells in the mucosa provide an opportunity to study the battlefield between the host and the virus and offers the potential of uncovering new insights about the pathogenesis and immunobiology of human HSV infections.

## MATERIALS AND METHODS

### Animals.

Nineteen mature (>5 years old), clinically healthy female RM (Macaca mulatta) with at least one term pregnancy and regular menstrual cycles were used in this study. All monkeys were selected from a specific-pathogen-free colony (SPF) that is seronegative for herpes B virus, simian T-cell lymphotropic virus type 1, simian retrovirus, and SIV. The serologic strategy for screening RM for herpes B virus infection has been described previously (31). All monkeys were housed in the California National Primate Research Center in accordance with the American Association for Accreditation of Laboratory Animal Care standards and were trained to receive vaginal swabs without sedation. The Institutional Animal Care and Use Committee of the University of California, Davis, approved all protocols in these experiments.

### HSV-2 isolates and vaginal HSV-2 inoculation.

Wild-type laboratory HSV-2 strains 186, 333, and G were used in this study because they have been widely used in mouse and guinea pig studies and they are pathogenic in these species (32–37). The HSV-2 186 strain was obtained from D. Knipe by D. Knoelle, and strain 333 was obtained from L. Corey. Both strains were minimally passaged (2 or 3 times) in Vero cells before expansion into a stock as described below. The HSV-2 G strain was purchased from the ATCC. Virus stocks of all HSV-2 strains were expanded in Vero cells and contained ∼1 × 10^7^ PFU/ml. To produce the stocks, supernatants from Vero cell cultures were harvested, pooled, and spun at 300 × *g* for 10 min to remove cellular debris. Clarified supernatants were aliquoted and frozen at −80°C until use. To inactivate HSV-2 for animal inoculations, aliquots of the stocks were pooled and heated to 56°C for 1 h and then frozen at −80°C overnight. This procedure was repeated 5 times for the HSV-2 pools used to expose animals 38044 and 37349 and 3 times for the HSV-2 pools used to expose animal 40257. We found that all the live stocks contained approximately 10^9.5^ vDNA copies/ml, while the DNA concentration in pooled killed HSV-2 stocks was higher (approximately 10^10^ vDNA copies/ml), presumably due to loss (sublimation/evaporation) of some media during the inactivation process.

For all intravaginal inoculations, 1 ml of total virus stock was introduced into the vaginal canal with a 1-ml needleless syringe while the animal was sedated and in sternal recumbency.

### Medroxyprogesterone acetate treatment.

As previously described (38), 4 weeks prior to and on the day of HSV-2 inoculation, medroxyprogesterone acetate (30 mg/animal) was administered by intramuscular injection to the 4 RM in group 3.

### *In vivo* depletion of CD8α^+^ lymphocytes (CD8^+^ T cells and NK cells).

Using methods previously described (39, 40), group 4 RM (*n* = 2) were treated with a CD8α^+^ lymphocyte (CD8^+^ T cells and NK cells)-depleting complementarity-determining region (CDR)-grafted rhesus IgG1 MAb, MT807R1, and the neutralizing CDR-grafted rhesus IgG1 anti-IL-15 MAb M111R1. The MT807R1 MAb was derived from a mouse anti-human CD8α MAb (41), and the M111R1 MAb was derived from a mouse anti-human anti-IL-15 MAb. The anti-IL-15 antibody prevents homeostatic proliferation of other lymphocyte subsets during CD8α^+^ lymphocyte depletion (42). Both MAbs (50 mg/kg of body weight) were administered by subcutaneous injection 3 days before and the day of HSV-2 inoculation. The NIH Nonhuman Primate Reagent Resource, University of Massachusetts Medical School (Boston, MA), provided the M111R1 and MT807R1 MAbs used in these studies.

### Collection of CVS samples, vaginal swabs, and tissues.

The schedule of genital tract collections is detailed in Tables S1 and S2. Postinoculation cervical sections were collected twice daily (6-h intervals), 5 days/week, by swabbing the ectocervix, vagina, and perineum. All monkeys were trained to accept twice-daily vaginal swabs with food rewards and without sedation. Two vaginal swabs were collected simultaneously twice daily from HSV-2 inoculation to necropsy. One swab was stored in UTM viral transport medium (Copan Diagnostics, Murrieta, CA) for virus isolation, while the other swab was stored in AVL buffer (Qiagen, Germantown, MD) for viral nucleic acid analysis. In addition, cervicovaginal secretion (CVS) samples were collected weekly by vigorously infusing 6 ml of sterile phosphate-buffered saline (PBS) into the vaginal canal and then aspirating as much of the instilled volume as possible while the RM were immobilized. Care was taken to ensure that the cervical mucus was included in the lavage fluid and that no trauma to the mucosa occurred during the procedure. Two-thirds of the CVS sample was snap-frozen on dry ice and stored at −80°C until analysis. The remainder was spun, and the supernatant was treated with 10× protease inhibitor (Roche) and subsequently used for cytokine and chemokine quantitation. The sample collection and preparation procedure resulted in at least a 10-fold dilution of the CVS. All samples were stored at −80°C until analysis. Genital tract tissues (vaginal mucosa, vulva, and perineum) and nervous tissues (including pelvic nerves, sacral sympathetic trunk, dorsal root ganglia, and sacral, lumbar, thoracic, and cervical spinal cords) were collected at the time of necropsy. Tissues were stored in RNAlater, which preserves all nucleic acids (Ambion, Austin, TX), and kept at −20°C until DNA extraction.

### Quantitative real-time PCR.

In brief, PCR to detect HSV-2 was performed in a validated quantitative PCR assay as previously described (43–46). DNA samples were prepared by using the QiaAmp DNA blood kit (Qiagen) according to the manufacturer’s instructions. Quantitative real-time PCR targeting HSV-2 gB DNA and HSV-2 polymerase DNA was used to determine the frequency and level HSV-2 DNA in vaginal swabs and tissues collected at necropsy. The cutoff utilized was 1 DNA copy/reaction, or 50 copies/μl of swab sample. To differentiate HSV-2 strains 186 and 333, we use primers and probes as follows: UL24 565F, GCATGTCTTTAGCTCCAGGATGA; UL24 565R, CCGCCCAGACTGCATCTG; UL24 565A, 6-carboxyfluorescein (FAM)-ATTGGCGAATTCAAA-MGBNFQ; and UL24 565G, VIC-TTGGCGAATTCGAA-MGBNFQ. We estimated cell number in a tissue sample by quantifying the glyceraldehyde-3-phosphate dehydrogenase (GAPDH) DNA copy number and dividing by 2. DNA genomes were measured with a qPCR primer-probe cocktail amplifying the human GAPDH gene (ABI). The PCR results are reported as HSV-2 DNA copies/swab for cervicovaginal swab samples, or HSV-2 DNA copies/million cells for tissue samples.

### HSV-2 isolation.

To isolate infectious HSV-2, eluents of vaginal swabs and CVS fluids were filtered, then added to human diploid fibroblasts and Vero cell cultures, and monitored for signs of HSV-2 replication as described previously (47).

### CVS proteome analysis.

Mass spectrometry was utilized to characterize host proteins in CVS as described previously (48, 49). CVS collected from macaques challenged with live (*n* = 12; groups 2, 3, and 4) and inactivated (*n* = 3; group 5) HSV-2 were analyzed in two independent mass spectrometry experiments. Briefly, 50 μg of protein from each sample was processed using filter-aided sample preparation, and isolated peptides were digested with trypsin. Peptides were cleaned of salts and detergents using reversed-phase liquid chromatography (high pH RP, Agilent 1200 series microflow pump, and Water XBridge column) and analyzed by label-free tandem mass spectrometry using an Orbitrap Q-Exactive mass spectrometer (Thermo Fisher Scientific, Waltham, MA). Mass spectrometry data were run through Progenesis QI software using default parameters (Nonlinear Dynamics, Durham, NC). Peptide identity searches were performed using Mascot Daemon v2.4.0 (Matrix Science, Boston, MA) against the UniprotKB/Swiss-Prot (2015) database, restricting taxonomy to both the *Homo* and *Macaca* genera. Host protein identifications were restricted to high-confidence hits using Scaffold (v4.4.1; Proteome Software, Portland, OR; confidence thresholds, ≤0.1% false-discovery rate [FDR] for peptide identification and ≤1% FDR for protein identification, ≥2 peptides identified). Significant changes in protein expression relative to prechallenge time points (day 0) were assessed using paired *t* tests and were adjusted for multiple-hypothesis testing using the Benjamini-Hochberg method. Functional enrichment analysis was performed using DAVID Bioinformatics Resources (6.7) against the Gene Ontology Database (50).

### Intracellular cytokine staining.

Flow cytometric intracellular cytokine analysis was used to detect HSV-specific CD8^+^ and CD4^+^ T-cell responses measured in cryopreserved splenocytes or PBMC preparations as previously described (51, 52). Briefly, isolated mononuclear cells were incubated with HSV lysate (HSV-2 isolates 186 and 333) or HSV gene-specific peptide open reading frame pools (ICP4, gD-1, UL25, and UL46; 15-mers overlapping by 11 at 2 μg/ml per peptide) and the costimulatory molecules CD28 and CD49d (BD Biosciences, San Jose, CA) alone for 1 h, followed by addition of brefeldin A (Sigma-Aldrich) for an additional 8 h. Costimulation with uninfected cell lysates (mock) served as a background control. The next day, stimulated cells were stained with fluorochrome-conjugated MAbs, including SP34-2 (CD3; Pacific Blue), L200 (CD4; AmCyan), SK-1 (CD8α; peridinin chlorophyll protein [PerCP]-Cy5.5), MAB11 (TNF-α; phycoerythrin [PE]), B27 (IFN-γ; allophycocyanin [APC]) FN50 (CD69; PE-Texas Red). Data were collected on an LSR-II (BD Biosciences). Analysis was performed using FlowJo software (Tree Star, Ashland, OR) and graphed using GraphPad Prism. In all cases, gating on the lymphocyte population was determined by the separation of the CD3^+^ T-cell subset and progressive gating on CD4^+^ and CD8^+^ T-cell subsets. Antigen-specific response frequencies for the CD4^+^ and CD8^+^ T-cell populations were determined from intracellular expression of either or both IFN-γ and TNF-α above background (mock).

### HSV-2-specific ELISA.

Plasma was also tested for HSV-2-specific antibodies with a whole-virus ELISA modified to detect nonhuman primate IgG. In brief, ELISA plates were coated with a 1:1 mixture of UV-inactivated, Vero cell-associated HSV-2 strain 186 and 333 lysates at a dilution of 1:200 each or control Vero cell lysate. After blocking, plasma samples were loaded for 2 h at room temperature. Controls for anti-HSV-2 binding included pre- and postvaccination mouse serum from prior studies (53), and controls for detection of macaque IgG were serum from animals before and after vaccination with vaccinia virus vector kindly provided by Shu-lock Hu, University of Washington. For the latter, a lysate of vaccinia virus grown in BSC-40 cells or control uninfected BSC-40 lysate was prepared as described previously (54) and used to coat ELISA plates. After washes, macaque IgG was detected with a 1:10,000 dilution of horseradish peroxidase (HRP)-conjugated affinity purified goat anti-monkey IgG (KPL, Gaithersburg, MD; part 074-11-021), while murine IgG was detected as described previously (53). After further washes, tetramethylbenzidine (TMB) substrate (KPL; part 50 to 76-00) was used per the manufacturer, reactions were stopped with phosphoric acid, and the optical density at 450 nm (OD_450_) was recorded. The detection of specific macaque IgG was confirmed by the detection of high levels of macaque anti-vaccinia virus IgG in sera from post- but not prevaccine sera from animals vaccinated with a vaccinia virus vector vaccine in a vaccinia virus antigen ELISA, with no binding to control BSC-40 cell antigen (data not shown). The antigenicity of the HSV-2 preparations used was confirmed by brisk binding of HSV-2-specific murine IgG to the HSV-2 antigen, with no binding to Vero cell control antigen (data not shown).

### Binding antibodies to HSV.

HSV antibodies in sera were also assessed with a Luminex-based assay. MagPlex microspheres (Luminex Corp.) were coated with 10 μg/ml of recombinant HSV-2 gD2, UL19, UL25, gJ, and ICP47 using an antibody coupling kit (Luminex Corp.) according to the manufacturer’s instructions and then blocked for a minimum of 1 h in 5% powdered milk–0.1% Tween 20–PBS. Primate plasma was then diluted 1:5, 1:25, and 1:125 in 1% milk–0.1% Tween 20–PBS assay buffer and incubated for 2 h with 2,500 each gD2, UL19, UL25, gJ, and ICP47 antigen-coated microspheres. Microspheres were washed three times in 0.1% Tween 20–PBS and then incubated with either a 1:500 dilution of anti-human IgG PE-conjugated secondary antibody (Southern Biotech; 2084-09) or 1:1,000 dilution of anti-monkey IgG biotin-conjugated secondary antibody (Nordic Immunological Laboratories, Tilburg, Netherlands) for 1 h. Microspheres were washed three times with 0.1% Tween 20–PBS. An additional incubation with 8 μg/ml of streptavidin-PE (Life Technologies; S-866) for 30 min followed by three washes with 0.1% Tween 20 PBS was performed for anti-monkey IgG secondary antibody microspheres. All microspheres were resuspended in 0.1% bovine serum albumin (BSA)–0.1% Tween 20–PBS and analyzed for antibody binding using a Luminex 200 instrument with MasterPlex CT software (Luminex Corp.).

### HerpeSelect 2 ELISA.

Heparinized plasma was tested for anti-HSV-2 glycoprotein G-specific IgG antibodies with a commercial kit, HerpeSelect 2 ELISA IgG (Focus Diagnostics), according to the manufacturer’s instructions, except that a peroxidase-conjugated goat anti-monkey IgG Fc polyclonal serum (Nordic Immunological Laboratories) was used as the secondary antibody.

### Immunohistochemistry/immunofluorescence.

To detect cell types and HSV-2 antigen-positive cells in tissue sections, immunohistochemistry or immunofluorescence was used. Tissue was snap-frozen in optimum cutting temperature compound (OCT) (Tissue-Tek; Sakura Finetek USA, Inc., Torrance, CA) and stored at −80°C until use. The frozen tissue was sectioned into 8-μm-thick slices for hematoxylin and eosin (H&E) histopathological analysis and immunofluorescence staining as described previously (30). Briefly, tissue sections were fixed and permeabilized in acetone before applying antibodies specific to HSV (rabbit polyclonal; Dako), CD4 (L200; BD Biosciences), and CD8 (RPA-T8; BD Biosciences). In dual staining, the tyramide signal amplification (TSA; Invitrogen) method was used for the CD4 antibody detection, followed by incubation with CD8α-Αlexa Fluor 647 (AF647) overnight. All tissue sections were counterstained with 4′,6-diamidino-2-phenylindole (DAPI; Fluka) before mounting in Mowiol 40 to 88 containing 2.5% (wt/vol) DABCO (Sigma-Aldrich, St. Louis, MO).

### Histopathologic analysis.

Tissues obtained by biopsy or necropsy were fixed with 4% paraformaldehyde, embedded in paraffin, and cut at 6 μm, and the sections were stained with hematoxylin and eosin. Slides were examined and micrographs made using a Zeiss Axiophot microscope.

### Data presentation and analysis.

Data were analyzed on Macintosh computers using Apple OSX10.11.6 and GraphPad Prism version 7 (GraphPad Software, San Diego, CA), and Macintosh computers (Apple Inc., Cupertino, CA) were used to graph the data.

### Statistical methods.

For comparing levels of HSV-2 DNA shedding between animal groups, the area under the curve (AUC) of the HSV-2 DNA level for each animal, defined as the average HSV DNA level over several intervals (0.5 to 28 days, 12 to 28 days, 28.5 to 39.5 days, and 0.5 to 39.5 days), was calculated. In addition, the HSV-2 DNA level decay rate over intervals (0.5 to 28 days and 28.5 to 39.5 days) separately was estimated for each animal based on fitting an exponential decay model for changes in DNA level over time. The exponential decay model assumes that the DNA level for an animal = α × 2^(–β^*^T^*^)^, where *T* is the time from the start of the time interval, β quantifies the decay rate, and 1/β quantifies the mean lifetime (i.e., at which the average DNA level is reduced to half its value at the beginning of the interval). The decay rate for each animal within an interval is computed using the nonlinear least square (nls) package (https://cran.r-project.org/ ). Both a two-sample *t* test and a Wilcoxon rank sum test (WRS) were used to test equality in distribution of AUCs and DNA level decay rates between animal groups.

## Supplementary Material

Supplemental file 1
