# Supplementary material for: Acute Infection and Subsequent Subclinical Reactivation of Herpes Simplex Virus 2 after Vaginal Inoculation of Rhesus Macaques
Source: J Virol. 2019 Jan 4;93(2):e01574-18. doi: 10.1128/JVI.01574-18 (PMC6321901; doi:10.1128/JVI.01574-18)
Supplement: Supplemental file 1 [file 94b75984269d77a0a12e4907dcf1e037_JVI.01574-18-s0001.pdf]

Supplemental Table 1. HSV-2 inoculation and sample collection schedule: Group 1

Days after HSV2 inoculation	Intervention/Sample						
	HSV inoculation	cervico-vaginal swab[a]	CVL[a]	Blood	cervical biopsy	vaginal biopsy	vulva/perineal biopsy
0	X	X	X	X	X	X	X
1		X	X	X			
2		X	X	X			
3		X	X				
4		X	X				
5							
6							
7	X	X	X	X	X	X	X
8		X	X				
9		X	X				
10		X	X				
11		X	X				
12							
13							
14	X	X	X	X			
15		X	X				
16		X	X				
17		X	X				
18		X	X				
19							
20							
21	X	X	X	X			
22		X	X				
23		X	X				
24		X	X				
25		X	X				
26							
27							
28		X	X	X			
29		X	X				
30		X	X				
31		X	X				
32		X	X				
33							
34							
35		X	X	X			
36		X	X				
37		X	X				
38		X	X				
39		X	X				
40							
41							
42		X	X	X			
43		X	X				
44		X	X				
45		X	X				
46		X	X				
47							
48							
49		X	X	X			
50		X	X				
51		X	X				
52		X	X				
54		X	X				
53							
55							
56	X	X	X	X			
57		X	X				
58		X	X				
59		X	X				
60		X	X				
61							
62							
63		X	X	X			
64		X	X				
65		X	X				
66		X	X				
67		X	X				
68							
69							
70		X	X	X			
71		X	X				
72		X	X				

a = collected twice daily in the Am and PM with an approximately a 6 hour interval

Supplemental Table 2. HSV-2 inoculation and sample collection schedule: Groups 2-5

Days after HSV2 inoculation	Intervention/Sample						
	HSV inoculation	cervico-vaginal swab[a]	CVL[a]	Blood	cervical biopsy	vaginal biopsy	vulva/perineal biopsy
0	X	X	X	X	X	X	X
1		X	X	X			
2		X	X	X			
3		X	X				
4		X	X				
5							
6							
7		X	X	X	X	X	X
8		X	X				
9		X	X				
10		X	X				
11		X	X				
12							
13							
14		X	X	X			
15		X	X				
16		X	X				
17		X	X				
18		X	X				
19							
20							
21		X	X	X			
22		X	X				
23		X	X				
24		X	X				
25		X	X				
26							
27							
28	X	X	X	X			
29		X	X				
30		X	X				
31		X	X				
32		X	X				
33							
34							
35		X	X	X			
36		X	X				
37		X	X				
38		X	X				
39		X	X				
40							
41							
42		X	X	X			
43		X	X				
44		X	X				
45		X	X				
46		X	X				
47							
48							
49		X	X	X			
50		X	X				

a = collected twice daily in the Am and PM with a 4 - 6 hour interval between samples

5

Supplemental Table 3. HSV-2 specific antibody responses in plasma collected from day 42-350 after HSV-2 inoculation.

Animal Number	Whole virus ELISA[a]	Western blot[b]	HerpeSelect 2 ELISA[c]	Luminex Assay[d]
34806	-	-	-	-
34889	-	-	-	+ [e]
35667	-	-	-	-
38420	-	-	-	-
33991	ND	ND	-	-
39406	ND	ND	-	+ [e]
33869	ND	ND	-	-
34099	ND	ND	-	-
35633	ND	ND	+	+[e]
37957	ND	ND	-	-
38775	ND	ND	-	ND
38769	ND	ND	-	ND
40371	ND	ND	+	ND
33993	ND	ND	-	ND
31602	ND	ND	-	ND
38953	ND	ND	-	ND

a = ELISA to detect IgG antibodies to UV-inactivated disrupted HSV-2 virions. After OD values from mock virus prep coated wells were subtracted from OD values of HSV-2 coated wells, there must be a 2 fold increase in OD from the pre-inoculation sample to the post inoculation sample (> 42 days PI) for an animal to be considered positive.
b = HSV-2 western blot to detect IgG antibodies to electrophoretically separated HSV-2 proteins. There must be 3 bands at appropriate locations in the blots incubated with the post inoculation plasma sample (> 42 days PI) for an animal to be considered positive.
c = HerpeSelect 2 ELISA to detect IgG antibodies to the glycoprotein G of HSV-2. There must be a 2-fold increase in OD from the pre-inoculation sample to the post inoculation sample (> 42 days PI) for an animal to be considered positive.
d= Luminex based assay to detect IgG antibodies to the glycoprotein J, glycoprotein D, UL25, UL19, and ICP47 of HSV-2 separately. There must be a 2 fold increase in MFI x dilution factor from the pre-inoculation sample to the post inoculation sample (> 42 days PI) for an animal to be considered positive.
e = The positive responses in all 3 animals was due to detection of anti-gD IgG antibodies

6
